# Characterizing behavioural differentiation in gene regulatory networks with representation graphs

**DOI:** 10.1093/nargab/lqae102

**Published:** 2024-08-09

**Authors:** Juris Viksna, Karlis Cerans, Lelde Lace, Gatis Melkus

**Affiliations:** Institute of Mathematics and Computer Science, University of Latvia, Raina bulvaris 29, Riga LV1459, Latvia; Institute of Mathematics and Computer Science, University of Latvia, Raina bulvaris 29, Riga LV1459, Latvia; Institute of Mathematics and Computer Science, University of Latvia, Raina bulvaris 29, Riga LV1459, Latvia; Institute of Mathematics and Computer Science, University of Latvia, Raina bulvaris 29, Riga LV1459, Latvia

## Abstract

We introduce the formal notion of representation graphs, encapsulating the state space structure of gene regulatory network models in a compact and concise form that highlights the most significant features of stable states and differentiation processes leading to distinct stability regions. The concept has been developed in the context of a hybrid system-based gene network modelling framework; however, we anticipate that it can also be adapted to other approaches of modelling gene networks in discrete terms. We describe a practical algorithm for representation graph computation as well as two case studies demonstrating their real-world application and utility. The first case study presents models for three phage viruses. It shows that the process of differentiation into lytic and lysogenic behavioural states for all these models is described by the same representation graph despite the distinctive underlying mechanisms for differentiation. The second case study shows the advantages of our approach for modelling the process of myeloid cell differentiation from a common progenitor into different cell types. Both case studies also demonstrate the potential of the representation graph approach for deriving and validating hypotheses about regulatory interactions that must be satisfied for biologically viable behaviours.

## Introduction

Hybrid system-based modelling frameworks have proven to be useful tools for describing and analysing gene regulatory systems (1). They provide a natural mechanism for integrating the discrete and continuous aspects characterizing a given biological system. From the perspective of simulating biological behaviour, they have the same power as differential equation-based models and have been (sometimes implicitly) used for such a purpose. At the same time, the discrete aspects of hybrid models allow reasoning about all the possible dynamic behaviours of the system in a similar manner to Boolean models, although the state spaces of hybrid models are considerably more complicated to analyse. Some generalizations of the Boolean framework (2,3) can actually be regarded as implicit hybrid systems and share similar state space structures.

Previously we have presented a specific rigidly defined hybrid system-based modelling framework HSM (4). The framework has been successful for phage virus modelling, allowing the reproduction of biologically known lytic and lysogenic cycles accurately and to prove that under certain constraints these two behaviours are the only stable states allowed for the modelled systems (5).

This promotes the HSM framework as a good candidate approach for modelling various differentiation processes in gene regulation. The framework by itself, however, remains very versatile, and its main strengths emerge from the analysis methods applied to the models. In this study, we focus on analysis methods and introduce the notion of representation graphs in formal terms. We further demonstrate the usefulness of the representation graph-based approach through two case studies.

The first case study considers previously developed phage virus models and re-analyses the behavioural dynamics of these models in terms of representation graphs. The second case study presents an HSM model of myeloid cell differentiation, derived from a simpler Boolean model (6). It shows how its representation graph captures evolutionary states leading to cell differentiation and gene activities that trigger transitions between these states.

For both of these case studies, we also demonstrate how the representation graph approach can be used for deriving and validating hypotheses about regulatory interactions, which need to be satisfied in order for the models to describe biologically viable behaviours.

## Materials and methods

### Modelling framework

For the description of gene regulatory networks (GRNs), we use a hybrid system-based HSM framework. Below we give a brief overview; for a detailed technical description, see (4). The definition of HSM is generally consistent with other widely accepted variations of hybrid system definitions, although it imposes some restrictions and simplifications—the goal is to keep the formalism as simple as possible whilst keeping it fully sufficient to describe the biological processes it intends to model.

HSM models are defined by tuples $\mathcal {H}=\left\langle M,X,C,T,F,MF \right\rangle$, where *M* = {μ_1_, …, μ_*k*_} is a set of modes; *X* = {*x*_1_, …, *x*_*m*_} is a set of continuous variables with real non-negative values; *C* = {*c*_1_, …, *c*_*r*_} is a set of real non-negative transition thresholds; *T* is a set of mode transitions in form τ = α → _*x*λ*c*_β, where α, β ∈ *M*, *x* ∈ *X*, *c* ∈ *C*, λ ∈ { ≤, ≥}), *F* = {*f*_1_, …, *f*_*n*_} is a set of continuous and monotonous functions; and *MF*: *M* × *X* → *F* is a mapping providing mode–function assignments assigning to each mode α ∈ *M* and each variable *x* ∈ *X* a function *g* ∈ *F*.

Intuitively, modes *M* represent states of the uneventful evolution of biological systems during which no observable events occur. Variables *X* describe concentration levels of biological substances (such as proteins) and *T* describes allowed transitions between the modes triggered by substance concentrations reaching specific thresholds or dropping below them. Functions from *F* describe changes of substance concentrations with time. For each substance, these changes are mode specific, and the behaviour at each of the nodes is specified by the mode–function assignment .

A distinctive aspect of our modelling framework is that we assume that a modelled biological system is fully defined by a particular HSM model $\mathcal {H}$, but our knowledge about the system is limited mostly by qualitative and not precise quantitative information (particularly regarding values of substance concentrations and their changes with time). Thus, essentially, we possess only some discrete knowledge about $\mathcal {H}$, which does not identify the exact model but only some wider class of models to which $\mathcal {H}$ belongs. In precise terms, such classes are described by the notion of HSM frames $\mathcal {F}(\mathcal {H})$, in which concrete functions from *F* are replaced by values from the set {↑, ↓} (we assume that for a particular state we know whether substance concentrations are increasing or decreasing) and our discrete knowledge about the system is represented by constraints *C*(*T*) defined by a partial ordering of transitions in *T*.

Such an assumption appears to be well justified from the perspective of experimentally measurable data that we can obtain about a specific biological system, with measurements usually being limited to comparative changes in substance (e.g. RNA or protein) concentrations and not their exact values. Also, unless we are mainly focused on the simulation of the modelled system under specific conditions [e.g. as in a hybrid system model developed for cardiac cell gene regulation (7)], there are few options apart from introducing and examining some type of discrete approximations of continuous state spaces inherent in hybrid system models.

Several techniques have been considered for building such discrete approximations, ranging from pre-defined partitioning of state spaces into regions representing different gene activity levels [thus, the state space of an *n* gene network is regarded as an *n*-dimensional hypercube (8,9)] to gradual step-wise discretization by introducing additional state space subregions during the process of backward reachability analysis from some final steady state (10).

To an extent, we adopt elements from both of these approaches. The discrete structure of frames $\mathcal {F}(\mathcal {H})$ is fully specified by the initial HSM, in which functions from *F* are treated as unknown, apart from the fact of whether they are increasing or decreasing, i.e. they could be replaced by either ↑ or ↓. The biological system is described by a class of models $\lbrace (\mathcal {F}(\mathcal {H}),C(T))\mid C(T) \in \mathcal {C}\rbrace$ satisfying constraints $C(T) \in \mathcal {C}$, with the initial set of constraints $\mathcal {C}$ incorporating the available biological knowledge. During the analysis phase, this class of models is gradually partitioned into smaller subclasses that represent observationally distinguishable behaviours of the modelled system.

### Describing gene regulatory networks with models

The HSM framework by itself does not directly associate any biological context with modes from the set *M* or with other model parameters; therefore, for useful biological models, such associations should be assigned by their design. The most natural (and effectively the only known) approach is to relate them in some way to gene activity. For example, in Boolean models, the states are directly defined by gene activity levels, assuming two such levels for each gene (either ‘active’ or ‘inactive’). In multivalued models, this assumption is further extended by allowing each of the genes to be in one of more than two discrete states (11).

A distinctive feature of our approach to model development is that we define the system’s HSM modes (‘states’) primarily by the occupancies of gene-binding sites rather than gene activity levels themselves. Correspondingly, the set of variables *X* represents proteins (products of genes included in the model), with 0 or more binding sites for each *x* ∈ *X*. This provides an important possibility to account for (biologically inherent) time delays for gene interactions—these delays fully depend on functions from *F* governing the substance change rates, although our knowledge about them remains limited by discrete constraints from *C*(*T*). Such a separation of gene and binding site activities has been introduced for finite state linear models (FSLMs) (12) and has been the key feature contributing to the modelling power that FSLM formalism provides. With the HSM framework having gradually evolved as a natural generalization of FSLMs, this also remains a key feature for the developed HSM models.

In line with the approach of model design focused on binding site activities, each binding site *B*[*x*] is assigned two real valued association and dissociation thresholds *a*(*B*[*x*]) and *d*(*B*[*x*]) with *d*(*B*[*x*]) < *a*(*B*[*x*]). A binding site-specific protein occupies the site *B*[*x*] when its concentration reaches *a*(*B*[*x*]); the site then becomes active. When the concentration of that protein drops below *d*(*B*[*x*]), the site becomes free and inactive. Using distinct binding site thresholds *d*(*B*[*x*]) < *a*(*B*[*x*]) is pivotal for model development, but intervals [*d*(*B*[*x*]), *a*(*B*[*x*])] are assumed to be too short to consider their partial overlaps. For two sites *B*_1_[*x*] and *B*_2_[*x*] binding the same protein, we assume that their affinities are either the same, (*B*_1_[*x*] = *B*_2_[*x*]), or one of them is strictly higher (*B*_1_[*x*] < *B*_2_[*x*])—such a type of constraint can be included in the initial model from the known experimental evidence or derived at the stage of model analysis. In most cases, each binding site will bind a specific protein. Still, occasionally it may be convenient to consider binding sites that can bind several competing binding factors—notably this is the case for phage virus models.

The set of modes *M* can be fully defined by binding site activities and the relationship of the current values of *X* variables to binding affinity constraints. Sets of modes with different relationships of *X* values to affinity constraints might be indistinguishable and can be merged, thus significantly reducing complexity. Two different modes, however, will necessarily have different binding site activity values. The set of transitions *T* is defined by Boolean functions, with their values dependent on binding site activity states. Each τ = α → _*x*λ*c*_β ∈ *T* switches activity of one particular gene, changing *MF*(*x*) from ↑ to ↓, or vice versa. For each α ∈ *M* and each *x* ∈ *X*, there is at most one transition from α labelled by *x*.

### Analysis of model dynamics

The dynamics of model $\mathcal {F}(\mathcal {H})$ are described by its state space graph. Let $\mathcal {F}(\mathcal {H})=\left\langle M,X,C,T,F,MF \right\rangle$. Its state space graph is an edge-labelled directed graph $G=(M,\hat{T})$ with the set of vertices *V*(*G*) = *M*, set of edges $E(G)=\hat{T}=\lbrace (\alpha ,\beta ) \mid \alpha \rightarrow _{x\lambda c}\beta \in T\rbrace$ and edge labels *l*((α, β)) = *x*.

To characterize all the possible dynamic behaviours the modelled system can exhibit, we are mostly interested in the following two properties of its state space *G*.

#### Regions of stable behaviour and attractors

Intuitively, stable behaviour regions should be characterized by sets of states *A*⊆*M* such that, once any state of *m* ∈ *A* is reached, the system will never reach any state outside *A*, and all the states from *A* will be revisited infinitely often. For Boolean models, such regions are known as attractors and are simple cycles.

In more complex state spaces, providing a precise definition for attractors that match these intuitive requirements becomes considerably more difficult. In the HSM framework, we define attractors as strongly connected components (SCCs), for which, additionally, the model does not imply that any trajectories entering these components will eventually leave them. Similar definitions of attractors have been proposed previously for several types of generalized Boolean models (3,13). For hybrid models, however, the state reachability problem is known to be algorithmically undecidable. Due to this, it cannot be guaranteed that two different stable behaviour regions will be separated into different attractors. Nevertheless, such a definition seems to work well for the models that we have analysed.

#### Definition

An induced subgraph *G*′ of *G* is an attractor, if: (i) *G*′ is strongly connected and (ii) there is no edge label *l*, such that for each state *m* ∈ *V*(*G*′) there is a transition (*m*, *m*′) with *l*((*m*, *m*′)) = *l*.

By *A*(*G*) we denote the set of all attractors in state space *G*.

#### Switching states and switching regions

We call *m* ∈ *M* a switching state if there is a transition $(m,m^{\prime })\in \hat{T}$, such that fewer attractors can be reached from *m*′ than from *m*. Typical model spaces can contain large numbers of switching states forming long and complex cascades, therefore it is useful to partition them into equivalence classes. Accordingly, we define switching regions as the maximum sets containing switching states of the same type.

#### Definition

*D*⊂*M* is a switching region if there exists a subset of attractors *S*⊆*A*(*G*), distinct proper subsets *S*_1_, …, *S*_*k*_⊂*S* and distinct labels *x*_1_, …, *x*_*k*_ ∈ *X*, such that: (i) for all *m* ∈ *D*, the set of all attractors reachable from *m* is *S;* (ii) for all *m* ∈ *D* and *i* = 1…*k*, there is a transition from *m* to *m*′ with *l*((*m*, *m*′)) = *x*_*i*_ and *S*_*i*_ being the set of all attractors reachable from *m*′; and (iii) *D* is the maximum set satisfying properties (i) and (ii).

By *D*(*G*) we denote the set of all switching regions in state space *G*.

### Representation graphs

As a convenient and concise way to describe the most informative features of model state spaces, we propose the formal notion of representation graphs. There are two motivating reasons for introducing them. Firstly, all the stable behaviours of the modelled system and the differentiation processes leading to them are largely characterized by the model’s state space attractors and switching regions; the internal structure of these regions is less significant. Secondly, an important observation from the models we have explored was that even very large state spaces (with tens of thousands of nodes) can contain only a few attractors and switching regions. Moreover, similar but different models tend to have the same sets of attractors and switching regions as well as transitions between them, although their state spaces are very different.

In representation graphs, each attractor *A*(*G*) and each switching region *R*(*G*) of a state space $G=(M,\hat{T})$ corresponds to a single vertex. In addition, we are interested in the dependence of attractor and switching state reachability on initial states. To characterize this, we consider a third type of regions, i.e. pool regions in state spaces, and assign a single vertex for each such region in *R*(*G*).

We say that *m* ∈ *M* is a pool state if $m\not\in U$ for any *U* ∈ *A*(*G*)∪*D*(*G*) (i.e. *m* is neither an attractor nor switching state) and *m* is not reachable from any switching state *m*′ (i.e. *m* does not lie on any path from a switching state to an attractor or another switching state).

#### Definition

*P*⊂*M* is a pool region if there is a set *S*⊆*A*(*G*)∪*D*(*G*) with labels *l*(*U*) ∈ *X* assigned to *U* ∈ *S*, such that: (i) for all *m* ∈ *P*, the set of all attractors and switching regions reachable from *m* is *S*; (ii) for all *m* ∈ *P*, *U* ∈ *S* and an edge (*m*′, *m*″) on a path from *m* to *U* with $m^{\prime }\not\in U$ and *m*″ ∈ *U*: *l*((*m*′, *m*″)) = *l*(*U*); and (iii) *P* is the maximum set satisfying properties (i) and (ii).

The single element set {*l*(*U*)} containing a label that defines reachability to *U* ∈ *S* we denote by *l*(*P*, *U*). By *P*(*G*),we denote the set of all pool regions in state space *G*.

According to these definitions, there can be no outgoing paths from attractors and no incoming paths to pool regions in *G*. Paths from switching regions to attractors and other switching regions are possible. For *U*_1_ ∈ *D*(*G*) and *U*_2_ ∈ *A*(*G*)∪*D*(*G*), we define set of labels *l*(*U*_1_, *U*_2_) = {*l*(*m*_1_, *m*_2_) ∣*m*_1_ ∈ U_1_, *m*_2_ ∉ *U*_1_, and (*m*_1_, *m*_2_) is an edge on a path from *U*_1_ to *U*_2_}. If *U*_2_ is an attractor reachable from *U*_1_, then *l*(*U*_1_, *U*_2_) will be a single element set.

Any node *m* ∈ *M* of state space *G* can belong to either an attractor, a switching region or a pool region, in which case *m* ∈ *U* for some *U* = *A*(*G*)∪*D*(*G*)∪*P*(*G*). We denote *r*(*m*) = *U*. Alternatively, a node can belong to neither of these three types of regions, in which case it lies on a path from a switching region to an attractor or another switching region. Due to a lack of a specific role in the modelled system’s dynamics, we treat such nodes as ‘non-interesting’ and do not include them directly in representation graphs.

#### Definition

The representation graph *R*(*G*) = (*V*, *E*) of $G=(M,\hat{T})$ is an edge-labelled directed graph with set of vertices *V* = *A*(*G*)∪*D*(*G*)∪*P*(*G*), set of edges *E* = {(*v*_1_, *v*_2_) ∣ *v*_1_, *v*_2_ ∈ *V*and there is a path from *r*(*v*_1_) to *r*(*v*_2_) in *G*} and edge labels $l((v_1,v_2))=l(r(v_1),r_(v_2))$.

Depending on a model, we might be interested only in the region of the state space reachable from a specific initial state (e.g. from 0-state with all binding sites being vacant), or the model by its design could be appropriate only for the description of the system dynamics starting from some particular state *m*. For example, the myeloid differentiation model described in our case study is designed to model only the differentiation phase after progenitor state CMP has already been reached. In such cases, the representation graph *R*(*G*′) can be constructed for the induced subgraph *G*′ with the vertex set of *G* restricted to those that are reachable from the node *m*.

Below we present an efficient and practical Algorithm 1 for representation graph computation. For a given HSM model frame’s state space $G=(M,\hat{T})$, the algorithm computes its representation graph *R*(*G*) = (*V*, *E*) and its set of attractors *A*(*G*). The internal structure of attractors (unless they represent single steady states) is of notable interest by itself, and for viable models it could be expected that all the attractors from *A*(*G*) can be assigned biologically feasible interpretations.

The algorithm starts by explicitly computing the set *A*(*G*) (finding SCCs is a well-known problem solvable in linear time). The computation of switching regions *D*(*G*) and pool regions *P*(*G*) is then done implicitly by performing vertex pair contractions in a copy of the input graph. Vertex contraction is performed by the function *Join*(*v*_1_, *v*_2_) that joins two graph vertices *v*_1_, *v*_2_ ∈ *V* of *G*(*V*, *E*) into a single vertex *v*_2_ and merges sets of incoming and outgoing edges from *v*_1_ and *v*_2_. Vertex label *l*(*v*_2_) is replaced by *l*(*v*_1_) ∪*l*(*v*_2_) and, for each edge *e* resulting from merging two edges *e*_1_ and *e*_2_, a new label *l*(*e*) = *l*(*e*_1_) ∪*l*(*e*_2_) is assigned. Initially assigned labels are single-valued; for notational simplicity, such single-valued *l* we also treat as one-element sets {*l*}.

Function *Join*(*S*) can also be applied to any non-empty subset of vertices *S*⊆*V* by iteratively calling *Join(v*_1_, *v*_2_) for *v*_1_, *v*_2_ ∈ *S* until *S* is reduced to a single vertex set {*v*}. Additionally, if there is a self-loop edge (*v*, *v*), then it is deleted. The function *Reach*(*S*) returns the union of the sets of vertices reachable from *v* ∈ *S*.


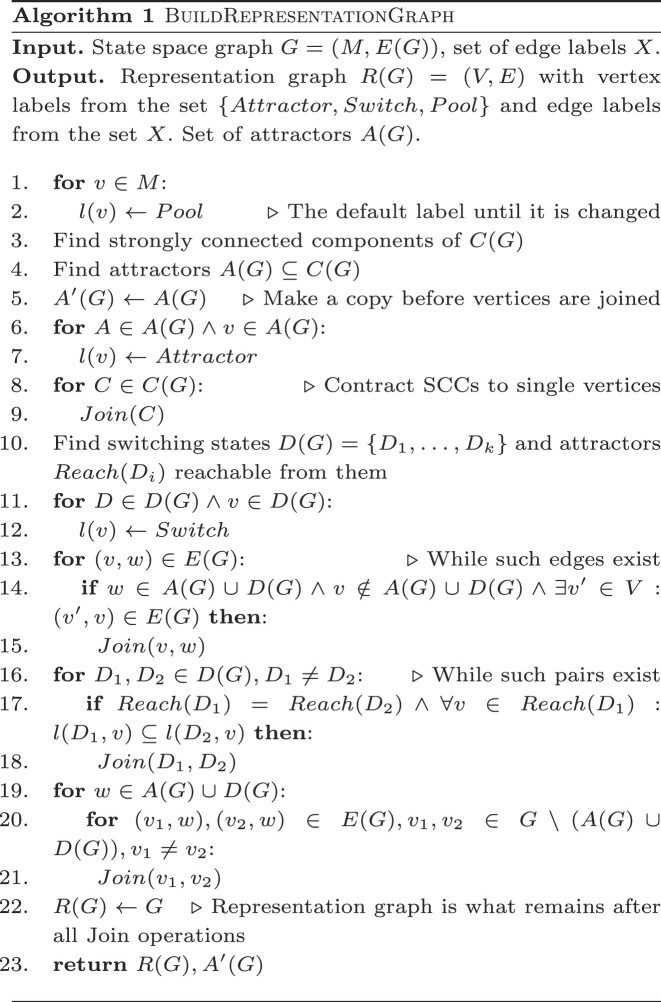


The algorithm initially computes SCCs and their reachability from other vertices in Θ(|*V*| + |*E*|) steps. Each of the remaining steps reduces the number of vertices by 1, thus there are at most (*O*|*G*|) of them, and, by performing *Join* operations in appropriately chosen order, this part of the algorithm can also be implemented in *O*(|*V*| + |*E*|) steps. To the algorithm’s time complexity, however, the sizes of edges labels |*l*(*e*)| and the sizes of sets of reachable vertices |*Reach*(*D*_*i*_)| also contribute.

|*l*(*e*)| is bounded by the number of genes *n*; the upper bound for the number of genes *n* in models for which the HSM framework could be applied is ∼30, and thus can be treated as constant. |*Reach*(*D*_*i*_)|, however, is bounded by *O*(|*A*(*G*)|) = *O*(|*V*|), and it is not difficult to construct artificial models with |*Reach*(*D*_*i*_)| = Θ(|*V*|), leading to *O*(|*V*|^2^ + |*E*|) worst case time complexity. For useful models, however, one should expect only a small number of attractors, in which case the time complexity becomes bounded by *O*(|*V*| + |*E*|). Thus, representation graphs can be effectively constructed for all HSM models for which state space graphs $G=(M,\hat{T})$ themselves are computable.

**Figure 1.**
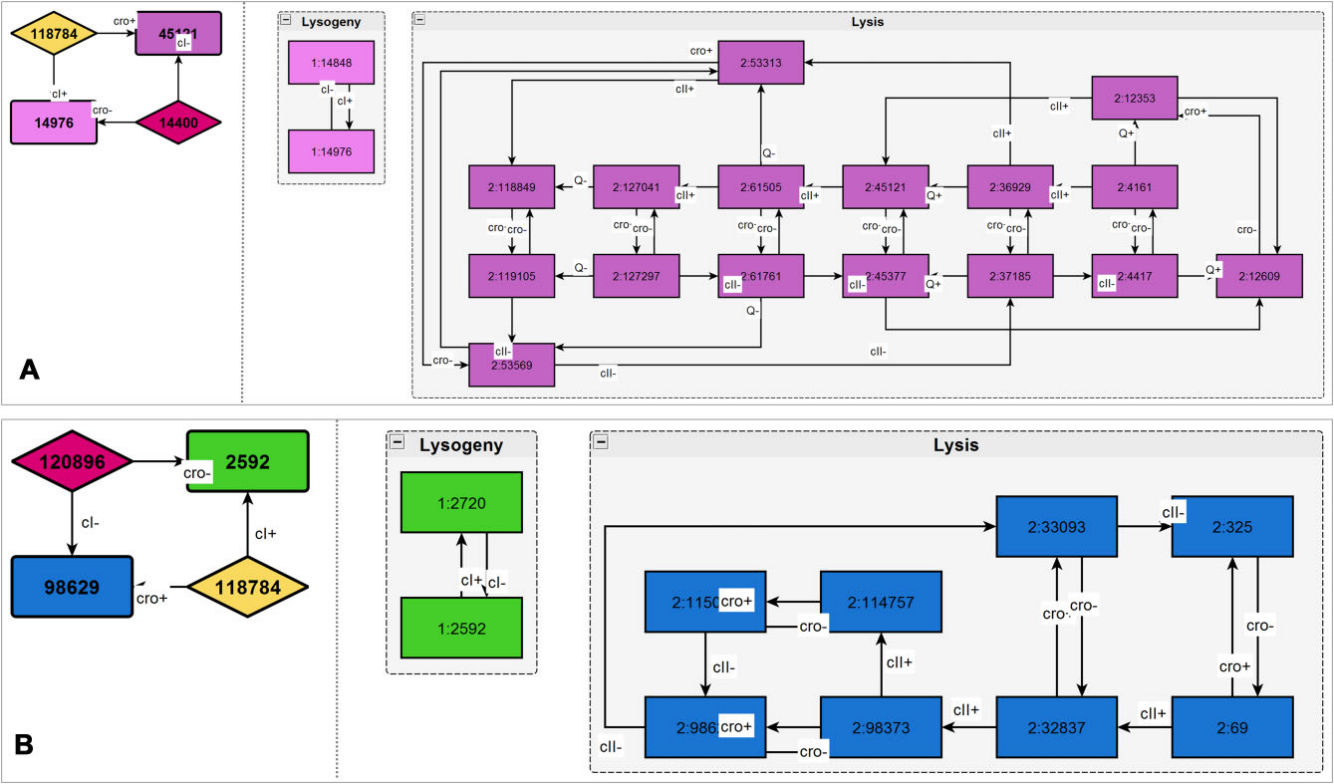
Representation graphs and the structure of lysis and lysogeny attractors for two lambda phage models. The visualizations are created with yEd diagram editor from GraphML output files by manually adjusting the layout and grouping vertices. In representation graphs (left), switching regions are shown by rhombi and attractors by coloured rectangles; pool regions are depicted by grey rectangles. The attractor graphs (right) show the corresponding parts of state spaces that are contracted in single attractor nodes in representation graphs. Only for model (**A**), lysis attractor corresponds to biologically feasible behaviour, whilst for model (**B**) it does not. Both models, however, have the same representation graph structure.

The algorithm is implemented in Python and is freely available online in GitHub and Zenodo repositories. The implementation relies on NetworkX (14) and N2G (15) libraries. The latter is used for an optional generation of representation attractor graph visualizations in GraphML format, which can be viewed by, e.g. yEd Graph Editor (16). Sample visualizations are shown in Figure 1 for two non-equivalent HSM frames of the lambda phage model. The representation graphs of both frames are identical (with pool states ignored). However, their attractor regions differ, with only a 16 state lysis attractor (Figure 1A), but not an 8 state lysis attractor (Figure 1B) being biologically feasible.

### Workflow of model design and analysis

**Figure 2.**
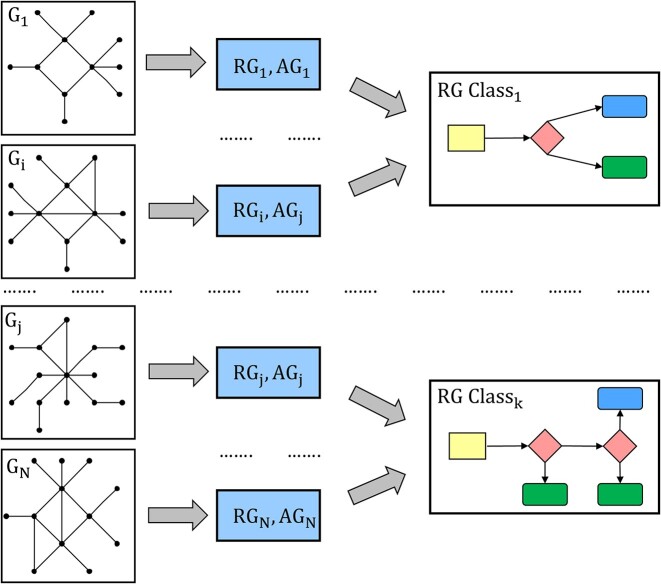
Workflow of HSM model analysis. A model is described by *N* state spaces, *G*_*i*_ corresponding to its HSM frames. From these we compute representation graphs *RG*_*i*_ and their attractor set *AG*_*i*_. Representation graphs are then further partitioned into equivalence classes defined by isomorphism.

A typical development of a HSM model starts by defining a model $\mathcal {H}$ with mode and transition sets built in accordance with a known gene regulatory mechanism and incorporating additional known biological assumptions as constraints on transition ordering. Such an initial model often has a trivial representation graph with few and non-meaningful attractors. The partial constraints placed in the model, however, can serve as a seed that can be complemented by additional hypothetical constraints. This provides a way to define and test many hypothetical models described by frames $\mathcal {F}_1,\ldots ,\mathcal {F}_N$, for which state space graphs *G*_*i*_ and the corresponding representation graphs *R*_*i*_ with their sets of attractors *A*_*i*_ are then being computed. A set of pairs (*R*_*i*_, *A*_*i*_) is then further partitioned into equivalence classes, with (*R*_*i*_, *A*_*i*_) and (*R*_*j*_, *A*_*j*_) defined to be equivalent if *R*_*i*_≅*R*_*j*_ and *A*_*i*_≅*A*_*j*_ (i.e. are isomorphic) as vertex- and edge-labelled graphs. For representation graphs, vertex labels distinguish between attractor, switching and pool states (and, depending on the focus of interest, pool states could be ignored). For attractors *A*_*i*_, their vertex labels are defined by occupancy states of binding sites. This analysis workflow is schematically shown in Figure 2.

## Results

We present two case studies that demonstrate the benefits of using representation graphs for analysing and understanding the different behaviours that biological systems described by HSM models can exhibit.

### Phage virus models

**Figure 3.**
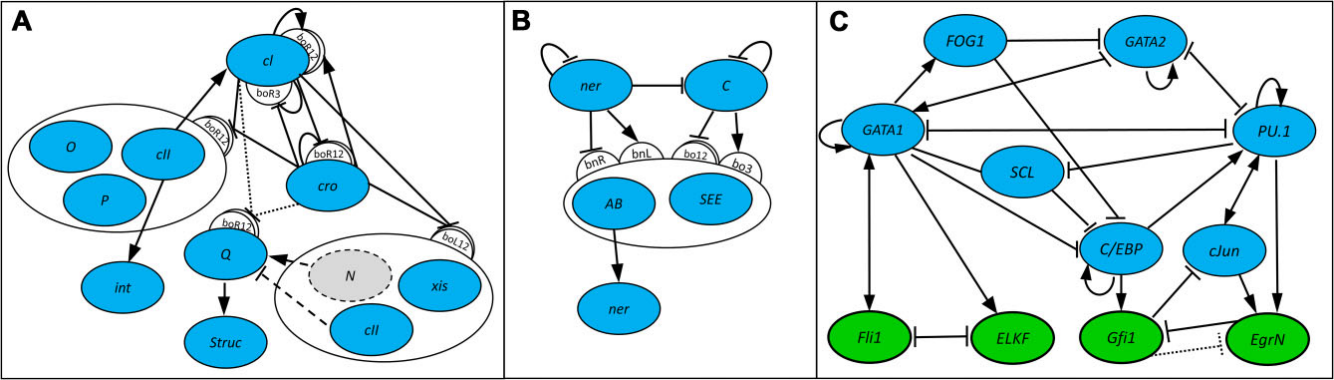
Schematic networks of gene interactions for lambda and HK022 phage (**A**), Mu phage (**B**) and myeloid cell differentiation (**C**) models. Sites binding two different transcription factors and sites binding the same transcription factor with opposite regulatory impacts on the regulated gene(s) are explicitly shown; otherwise, the presence of a single binding site is implicitly assumed for each regulatory interaction. Lambda and HK022 phage models (A) differ by regulation of gene *Q*, in lambda phage *Q* being regulated by *N* and *cII* (as shown with dashed lines), and in HK022 phage being regulated by *cro* and *cI* (as shown with dotted lines); in HK022 phage, gene *N* is absent. For the myeloid differentiation model (**C**), reaching one of the four steady states is fully determined by activation of one of the genes *Fli*-1, *ELKF*, *Gfi*-1 or *EgrNab*. In a model variant, direct inhibition of *EgrNab* by *Gfi*-1 is absent.

The first case study covers three bacteriophage virus models (Figure 3), lambda phage (model LPH2), HK022 phage and Mu phage. Detailed descriptions of these models and their design considerations are discussed in (17).

The development of phage models was driven by the availability of a very comprehensive biological description of gene regulatory processes of lambda phage by McAdams and Shapiro (18). This semi-formal description was used by Brazma and Schlitt (12,19) as a basis for proposing already a strictly formalized lambda phage model described by a FSLM framework. A notable achievement of the model and the proposed FSLM framework was the possibility of obtaining very realistic simulations of the two known characteristic behaviours of lambda phages: lytic and lysogenic cycles. The modelling power needed to accomplish this largely stems from introduction of explicit separation between genes and transcription factor-binding sites and their activities. A limiting feature of FSLM, however, is the assumption that protein concentration changes are described by linear functions, which could be considered an oversimplification from a biological perspective.

For the particular lambda phage model, however, it was also noted that the exact forms of functions governing protein concentration changes have limited importance. At the same time, the monotonicity of these concentration change functions is what matters for simulated behaviours to be consistent with biological observations. This observation was one of the driving factors for developing HSM as a more general modelling framework, which allows much greater flexibility for the choice of concentration change functions and also inherits a convenient way of redefining FSLM models in terms of the HSM framework. Analysis of the LPH1 lambda phage model thus obtained showed that lysis and lysogeny are the only stable behaviours possible according to the model. It also allowed the formulation of several hypotheses about comparative levels of binding site affinities that must hold for the model to describe biologically feasible lytic and lysogenic behaviours (5).

Here we present a revised and slightly more complex LPH2 model (Figure 3A) of lambda phage virus, which incorporates more recent biological knowledge (20). In particular, it introduces an extra binding site for one of the proteins and proposes somewhat different regulation mechanisms for a couple of the involved genes. The model contains 11 proteins and their corresponding genes: cI, cII, cIII, cro, int, N, O, P, Q, xis and Struc (denoting a set of co-regulated structural proteins), and 11 binding sites: bcII-1, bcII-2, bcII-3 (binding cII), bN (binding N), bQ (binding Q), and operator OR and OL binding sites bOR1, bOR2, bOR3, bOL1, bOL2 and bOL3 (each of these sites is competitively binding proteins cI and cro). The dependence of gene activity states from binding site occupancies is defined by the following Boolean formulae (with bS being true for an occupied binding site, and bS[P] being true for binding site occupied specifically by protein P):


\begin{eqnarray*} \begin{array}{l}{\rm cI}=\lnot {\rm bOR3} \wedge \\ ({\rm bOR1[cI]} \vee {\rm bOR1[cro]} \vee {\rm bOR2[cI]} \vee {\rm bOR2[cro]} \vee {\rm bcII}\hbox{-}1),\\ {\rm cro,cII,O,P}=\lnot {\rm bOR1} \wedge \lnot {\rm bOR2},\\ {\rm Q}={\rm bN} \wedge {\rm bcII}\hbox{-}3,\\ {\rm N,cIII,xis}=\lnot {\rm bOL1} \wedge \lnot {\rm bOL2},\\ {\rm int}={\rm bcII}\hbox{-}2,\\ {\rm Struc}={\rm bQ}. \end{array} \end{eqnarray*}

In terms of the HSM framework, LPH2 is described by a frame with the set of modes M almost being defined by all different occupancy states of the binding sites (up to 23 328 modes, the exact number depends on binding site affinity constraints), and with each variable from the set *X* representing one of 11 proteins. The Boolean formulae of gene activities define a set of transitions *T* and mode–function assignments *MF*.

The biologically known constraints of binding site affinities for the LPH2 model are the following: bcII-2 < bcII-1, bOR1[cI] < bOR2[cI] < bOR3[cI], bOL1[cI] < bOL2[cI] < bOL3[cI], bOR3[cro] < bOR2[cro] < bOR1[cro] and bOL3[cro] < bOL2[cro] < bOL1[cro], with comparative affinities between bOR and bOL operator sites and for the bcII-3 site being unknown. These constraints lead to 1200 different hypothetical HSM frames to be considered during the model analysis phase.

The HK022 model (Figure 3A) is based on general biological knowledge (21,22) and is broadly similar to LPH2 but has a slightly different regulatory mechanism. Notably, however, HK022 does not have antitermination gene *N*, and the lack of it has been considered as a difficulty to fully understand how its regulatory mechanism behaves. The model includes 10 proteins and their corresponding genes: cI, cII, cIII, cro, int, O, P, Q, xis and Struc, and nine binding sites: bcII-1, bcII-2, bQ, and operator OR and OL binding sites bOR1, bOR2, bOR3, bOL1, bOL2 and bOL3. The gene activities are defined by the following Boolean formulae:


\begin{eqnarray*} \begin{array}{l}{\rm cI}=\lnot {\rm bOR3} \wedge \\ ({\rm bOR1[cI]} \vee {\rm bOR1[cro]} \vee {\rm bOR2[cI]} \vee {\rm bOR2[cro]} \vee {\rm bcII}\hbox{-}1),\\ {\rm cro,cII,O,P,Q}=\lnot {\rm bOR1} \wedge \lnot {\rm bOR2},\\ {\rm cIII,xis}=\lnot {\rm bOL1} \wedge \lnot {\rm bOL2},\\ {\rm int}={\rm bcII}\hbox{-}2,\\ {\rm Struc}={\rm bQ}. \end{array} \end{eqnarray*}

The known constraints of HK022 binding site affinities are the same as those of the lambda phage LPH2 model, which leaves for analysis 400 different hypothetical HSM frames with up to 5832 modes.

The Mu model (Figure 3B) is based on the biological description of its regulatory network by Paolozzi and Ghelardini (23). The virus exhibits a similar type of lytic and lysogenic behaviour to lambdoid phages; however, the genes and regulatory mechanisms involved are very different. The model includes five proteins and their corresponding genes: c, ner, SEE, trsp and AB (denoting two co-regulated and co-binding proteins A and B), and five binding sites: bNL, bNR (binding ner), bO2, bO3 (binding c) and bIAS (binding AB). The gene activities are defined as follows:


\begin{eqnarray*} \begin{array}{l}{\rm c}=\lnot {\rm bO3} \wedge \lnot {\rm bNL},\\ {\rm ner}=\lnot {\rm bO2} \wedge \lnot {\rm bNR},\\ {\rm AB,SEE}=\lnot {\rm bO2} \wedge \lnot {\rm bNR} \wedge {\rm bNL},\\ {\rm trsp}={\rm bIAS}. \end{array} \end{eqnarray*}

The known binding site affinity constraint is bO2 < bO3; comparative affinities of bNL and bNR sites are unknown. This gives for analysis two HSM frames with up to 32 modes in each.

**Figure 4.**
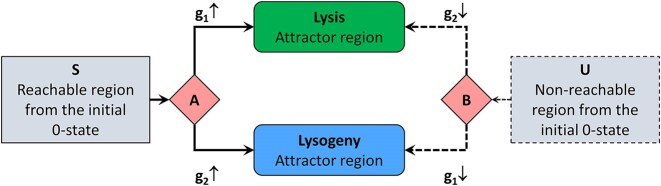
Representation graph describing state spaces of all phage virus models (lambda, HK022 and Mu phages). All models have exactly two attractors that can be associated with lysis and lysogeny and exactly two switching regions: A and B. The attractor reachability depends on comparative activity levels of two genes *g*_1_ and *g*_2_ (the involved genes differ for lambdoid and Mu phages). Depending on the model, the pool regions of non-switching states S and U could also be empty.

Comprehensive analysis of the individual models is presented in (17), but here we would like to highlight the fact that, apart from labels that correspond to gene names, the representation graphs for all three phage virus models and all the frames defining them are the same. The phage representation graph is shown in Figure 4. All models have exactly two attractors that can be associated with lysis and lysogeny and exactly two switching regions: A and B. The attractor reachability depends on comparative activity levels of two genes *g*_1_ and *g*_2_. The genes *g*_1_ and *g*_2_, whose activity irrevocably switches the models to lytic and lysogenic behaviours, are *cI* and *cro* for LPH2 and HK022, and *c* and *ner* for the Mu model.

Although all 1200 LPH2 frames share the same representation graph shown in Figure 4, they partition into six equivalence classes with the different topologies of lysis and lysogenic attractors, and only two of these classes (one including 380 and the other 190 frames) can be considered biologically feasible. The inclusion in one of these two classes requires an additional affinity constraint bOR3[cI] < bOL1[cI] for valid lysogeny behaviour, and a bOR2[cro] < bOL2[cro] constraint for valid lysis behaviour. These affinity constraints can be viewed as hypotheses predicted by the model and, in principle, could be validated experimentally, although such validation remains complicated. In both of these two classes, lysogeny attractor regions contain two states, and lysis attractor regions contain 16 states, with transitions in the lysis attractor region being different for each class. The exact type of lysis attractor depends on affinity constraints on cII binding, with the constraint bcII-2 < bcII-3 < bcII-1 leading to one of these lysis types and both alternative constraints bcII-3 < bcII-2 < bcII-1 and bcII-2 < bcII-1 < bcII-3 leading to another.

The default assumption for phage virus models is that, after the initial infection of bacteria, all binding sites should be vacant (with virus proteins not yet present). Thus, of clear relevance are model state space regions reachable from the initial 0-state. In representation graphs of lambdoid phage models, these state space parts are covered by the switching region A (Figure 4) and two attractor states, with an irrevocable transition to either lysis and lysogeny behaviours depending on competitive growth rates of cI (g_1_) and cro (g_2_) proteins.

However, a topic of increasing research interest is also infection of a single cell by multiple phage viruses (24–26), in which case reachability from other states and not only the 0-state can become relevant, i.e. the switching region B and the pool region U could also have active biological roles. In principle, the complete state spaces and their representation graphs of our LPH2 model are expected to fully characterize all the possible behaviours in such multiple infection scenarios (thus, lysis–lysogeny switching could also occur while concentrations of cI and cro decrease). Admittedly, the current model might not fully cover biological interactions that become significant when multiple copies of lambda phage are present within a single cell.

Similarly, all 400 HK022 frames share the same representation graph of Figure 4. All HK022 frames are contained in a single equivalence class with two attractors that can be well associated with feasible lysis (described by a two state attractor) and lysogeny (described by a 12 state attractor) behaviours (17). Such a result, whereby all HK022 model frames behave in a very similar and biologically feasible way, independently of additional constraints, is somewhat unexpected, given that the lack of N protein is considered problematic for the model’s stability.

For the Mu model, we have two initial frames, from which only the frame satisfying affinity constraint bNL < bNR has two attractor regions that can be well associated with lysis (two state attractor) and lysogeny (four state attractor). The representation graph again is the same as shown in Figure 4, although in this case proteins triggering the lysis–lysogeny switch are completely different—c (g_1_) and ner (g_2_) (17). The alternative affinity constraint bNL > bNR leads to a state space with a single attractor without lysis–lysogeny differentiation, thus the constraint bNL < bNR is a prediction obtained from the model, which could be validated experimentally.

### Myeloid progenitor differentiation model

The second case study describes myeloid cell differentiation in a mouse (used as a model organism due to data availability, with expectations that the model can be adapted and transferred to other mammalian organisms, including humans). It is based on a very detailed Boolean model (6) describing a tree-like evolutionary process of divergence of common myeloid progenitors (CMPs) into granulocyte–monocyte progenitors (GMPs) and megakaryocyte–erythrocyte progenitors (MEPs) that further evolve into granulocytes, monocytes, megakaryocytes and erythrocytes. The model involves 11 genes, and the authors provide a very detailed assessment of a particular choice of regulatory functions with varying degrees of underlying evidence for the included regulatory interactions and functions—ranging from experimentally confirmed regulatory interactions to co-related gene expression and some hypothetical interactions. The dynamics analysis assumed a fully asynchronous model behaviour, and the authors have identified the main genes contributing to specific evolutionary choices. However, the paper does not provide enough details for the full reproducibility of the results.

Our base HSM model of myeloid cell differentiation (Figure 3C) has been derived from the Boolean model (6) and contains 11 proteins and their corresponding genes: GATA-1, GATA-2, FOG-1, EKLF, Fli-1, SCL, C/EBPα, Pu.1, cJun, EgrNab and Gfi-1. The total number of the involved binding sites is 30 (including some that are not confirmed experimentally but implied from regulatory assumptions), with multiple binding sites for most of the proteins. However, due to a lack of evidence that affinities of different sites binding the same gene might differ, for modelling purposes we include only a single binding site bG for each gene G. The gene activities are defined by the same formulae as in the Boolean model (6), substituting for function arguments the activity states of the corresponding gene-binding sites:


\begin{eqnarray*} \begin{array}{l}{\rm GATA}\hbox{-}1 = ({\rm bGATA}\hbox{-}1 \vee {\rm bGATA}\hbox{-}2 \vee {\rm bFli}\hbox{-}1) \wedge \lnot {\rm bPU.1},\\ {\rm GATA}\hbox{-}2 = \lnot ({\rm bGATA}\hbox{-}1 \wedge {\rm bFOG}\hbox{-}1) \wedge {\rm bGATA}\hbox{-}2 \wedge \lnot {\rm bPU.1},\\ {\rm FOG}\hbox{-}1 = {\rm bGATA}\hbox{-}1,\\ {\rm EKLF} = {\rm bGATA}\hbox{-}1 \wedge \lnot {\rm bFli}\hbox{-}1,\\ {\rm Fli}\hbox{-}1 = {\rm bGATA}\hbox{-}1 \wedge \lnot {\rm bEKLF},\\ {\rm SCL} = {\rm bGATA}\hbox{-}1 \wedge \lnot {\rm bPU.1},\\ {\rm C/EBP}\alpha = {\rm bC/EBP}\alpha \wedge \lnot ({\rm bGATA}\hbox{-}1 \wedge {\rm bFOG}\hbox{-}1 \wedge {\rm bSCL}),\\ {\rm PU.1} = ({\rm bC/EBP}\alpha \vee {\rm bPU.1}) \wedge \lnot ({\rm bGATA}\hbox{-}1 \vee {\rm bGATA}\hbox{-}2),\\ {\rm cJun} = {\rm bPU.1} \wedge \lnot {\rm bGfi}\hbox{-}1,\\ {\rm EgrNab} = {\rm bPU.1} \wedge {\rm bcJun} \wedge \lnot {\rm bGfi}\hbox{-}1,\\ {\rm Gfi}\hbox{-}1 = {\rm bC/EBP}\alpha \wedge \lnot {\rm bEgrNab}. \end{array} \end{eqnarray*}

The model describes the differentiation of CMPs into granulocyte, monocyte, megakaryocyte and erythrocyte cell types. Reaching steady states corresponding to these cell types is fully determined by activation, correspondingly, of one of the genes *Gfi*-1, *EgrNab*, *Fli*-1 and *ELKF*. To ensure the stability of these steady states, the model includes explicit inhibitory regulations between *Gfi*-1, *EgrNab* and *Fli*-1, *ELKF* gene pairs, although experimental evidence supporting these inhibitory regulations is partial and indirect. In particular, it has been noted that direct inhibition of *EgrNab* by *Gfi*-1 might not be strictly necessary (6), and our HSM model is well suited for testing such a hypothesis. We do this by considering a model variant where this inhibitory regulation is absent, and *EgrNab* regulation is given by the formula:


\begin{eqnarray*} {\rm EgrNab} = {\rm bPU.1} \wedge {\rm bcJun}. \end{eqnarray*}

(Similarly, we have tested the need for direct inhibitions of *Gfi*-1, *Fli*-1 and *ELKF* genes; however, these were shown to be necessary for differentiation, according to the model, to occur.)

**Figure 5.**
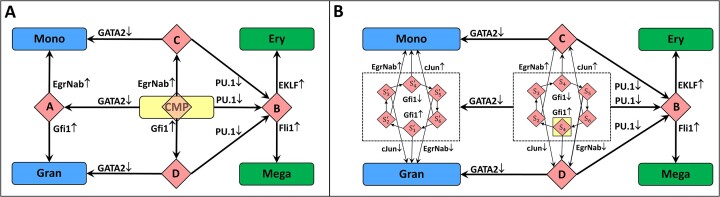
Representation graphs for the myeloid cell differentiation model. (**A**) Base model with explicit inhibitory gene interactions preventing transitions between granulocyte–monocyte and megakaryocyte–erythrocyte state pairs as soon as one of these steady states has been reached. (**B**) A model variant without explicit inhibition of the *EgrNab* gene by *Gfi-1*. The model still provides complete separation between steady states corresponding to granulocyte and monocyte cell types, although differentiation paths to these states from CMPs become more complex. Single switching states CMP and A are replaced by six state cycles; transition to and from dotted rectangles containing these cycles apply to the all states contained in them. Notably, both models have regions (B) that can be well associated with MEP states, but there are no regions that unambiguously can be associated with GMP states.

The representation graph of the base model is shown in Figure 5A. By the model design, all attractors are intended to be single-state regions, thus of primary interest are switching states and trajectories from the CMP state leading to four attractor regions. The model adequately shows the evolution of CMPs into four different cell types and confirms the presence of a well-defined intermediate MEP region (region B). There is no clearly defined GMP region, however, which remains divided into three parts: A, C and D, from which only A can be viewed as a GMP, while from C and D regions there remain evolution paths to the MEP region. This seems consistent with some recent work (27) indicating that the absence of a clearly defined GMP region might align with experimental observations. However, the competitive concentration drops of GATA-1 and PU.1 are decisive for the differentiation of CMP into granulocyte–monocyte and megakaryocyte–erythrocyte subtypes.

The representation graph of the model variant lacking *EgrNab* inhibition by Gfi-1 is shown in Figure 5B. It confirms the hypothesis that direct *EgrNab* inhibition might not be necessary for differentiation into stable granulocyte and monocyte cells, although differentiation paths to these states from CMPs become more complex. Parts of the base model representation graph corresponding to CMP and A switching states in the model variant are replaced by two six state cycles S_1_, …S_6_ and ${\rm S}_1^\prime ,\ldots {\rm S}_6^\prime$ with identical regions reachable from all the states of these cycles, albeit with different transition conditions to them. The biological accuracy of such cyclic switching trajectories is disputable; however, their presence relies on a number of other unverified assumptions incorporated into the model, and the fact that direct *EgrNab* inhibition might be unnecessary appears to be generally confirmed by the model.

It should be noted that the Boolean model (6), as well as our HSM model derived from it, are explicitly designed to describe only the differentiation phase that starts from the CMP state already being reached and are not intended to describe biological processes leading to CMP. Accordingly, the representation graphs in Figure 5 cover only parts of state spaces reachable from CMP as the initial state.

It might seem tempting to introduce multiple binding sites for some of the genes in our myeloid cell differentiation model (especially where there is good experimental evidence for their existence); however, there is a lack of objective biological evidence to justify this. Moreover, in comparison with virus models, the regulatory network is much more densely interconnected, and most of the genes are directly involved in regulatory activities; it can be checked that by introducing binding affinity constraints only as ’hypotheses for verification’ one can quite easily custom design models that have the desired behaviours but lack biological relevance. More generally, this also highlights the differences between using a HSM-based approach for the modelling of small and compact virus gene networks, when we can aim for the development of a model for the entire ‘life cycle’ of a particular biological process, in comparison with modelling much larger strongly interlinked eukaryotic gene networks, where we are confined to the development of models for only a small part of a biological system.

## Discussion

The main goals of the study were the introduction in formalized and well-defined terms of the concept of representation graphs, and the demonstration of the utility of this formalized notion for analysing behaviour dynamics of GRN models. The two presented case studies show the particular suitability of representation graphs for the characterization of processes that lead to irrevocable differentiation of the modelled system’s behaviour into several distinct regions of stability.

The first of the case studies is based on our previous work on phage virus modelling. The introduction of the concept of representation graphs has been strongly influenced by informal-style visualizations that we have used to illustrate the phage models’ behaviours. However, the definition of this notion in a formal and rigid manner allows these results to be captured in a compact and uniform way that provides a precise understanding of the evolutionary dynamics the models allow. In particular, the realization that all viable phage virus models share the same representation graph describing their state space structures was unanticipated before these compact representations were obtained.

The second case study proposes a redefinition of the known Boolean model of myeloid cell differentiation in terms of the HSM framework. Besides showing the advantages of this framework for model description, it very well demonstrates the merits of representation graphs for providing a detailed understanding of differentiation processes that govern the evolution of a progenitor state cell into one of the four mature cell subtypes. Both of these case studies also show the capacity of the modelling framework to suggest and validate new hypotheses about regulatory interactions.

The presented algorithm for computing representation graphs from state spaces of the modelled systems essentially shows that their use for analysing the models’ behaviour is practical as long as the models’ state spaces can be computed efficiently. The size of state spaces grows exponentially with *n* (in the case of HSM, *n* denotes the number of the modelled protein-binding sites). This limits the upper size of the models to which HSM framework- and/or representation graph-based analysis can be effectively applied to *n* ∼ 30; the value is similar to the upper size of Boolean GRN models that can still be fully analysed.

By their definition, the application of representation graph-based behaviour analysis is meaningful only for models that describe some process of differentiation in which the system can evolve to more than one stable state; otherwise their representation graphs are trivial. Admittedly, the number of known or published GRN models (including those presented in not particularly rigid terms) of such a type remains rather limited. Nevertheless, for models describing some kind of differentiation process, the representation graphs will provide answers to the most natural questions about the model’s behaviour that one might ask. As far as we are aware, apart from the case of simple Boolean GRN models, no comparable formalized methods for comprehensive analysis of the structure of state spaces of the modelled biological systems have been proposed before.

As they are used here, the representation graphs are specific to a particular hybrid system-based modelling framework. However, as the example of myeloid cell differentiation models shows, there appears to be a good potential to adapt them to other GRN modelling approaches in which the networks can be described in terms of discrete state spaces.

## Data Availability

Open source Python implementation of the *BuildRepresentationGraph* algorithm is freely available on Zenodo at https://doi.org/10.5281/zenodo.10851071 and on GitHub at https://github.com/IMCS-Bioinformatics/HybridSystemModels-RepresentationGraphs. The repository also includes all datasets—state space graphs for phage virus and myeloid cell differentiation models as well as their representation graphs and attractor regions as computed by the algorithm.

## References

[1] Glass L., Edwards R. Hybrid models of genetic networks: mathematical challenges and biological relevance. J. Theor. Biol. 2018; 458:111–118. 30227116 10.1016/j.jtbi.2018.09.014

[2] Siebert H., Bockmayr A. Temporal constraints in the logical analysis of regulatory networks. Theor. Comput. Sci. 2008; 391:258–275.

[3] Serra R., Vilani M., Barbieri A., Kaufmfman S., Colacci A. On the dynamics of random Boolean networks subject to noise: attractors, ergodic sets and cell types. J. Theor. Biol. 2010; 265:185–193. 20399217 10.1016/j.jtbi.2010.04.012

[4] Brazma A., Cerans K., Ruklisa D., Schlitt T., Viksna J. Modeling and analysis of qualitative behavior of gene regulatory networks. Lect. Notes Comput. Sci. 2015; 7699:51–66.

[5] Ruklisa D., Brazma A., Cerans K. T. S., Viksna J. Dynamics of gene regulatory networks and their dependence on network topology and quantitative parameters—the case of phage lambda. BMC Bioinformatics. 2019; 20:296. 31151381 10.1186/s12859-019-2909-z PMC6544977

[6] Krumsiek J., Marr C., Schroeder T., Theis F. Hierarchical differentiation of myeloid progenitors is encoded in the transcription factor network. PLoS One. 2011; 6:e22649. 21853041 10.1371/journal.pone.0022649 PMC3154193

[7] Grosu R., Batt G., Fenton F., Glimm J., Le Guernic C., Smolka S., Bertozzi E. From cardiac cells to genetic regulatory networks. Lect. Notes Comput. Sci. 2011; 6806:396–411.

[8] Edwards R. Analysis of continuous-time switching networks. Phys. D. 2000; 146:165–199.

[9] Edwards R., Glass L. A calculus for relating the dynamics and structure of complex biological networks. Adv. Chem. Phys. 2005; 132:151–178.

[10] Ghosh R., Tomlin C. Symbolic reachable set computation of piecewise affine hybrid automata and its application to biological modelling: Delta-notch protein signalling. Syst. Biol. 2004; 1:170–183. 10.1049/sb:20045019 17052127

[11] Thomas R. Regulatory networks seen as asynchronous automata: a logical description. J. Theor. Comput. Biol. 1991; 153:1–23.

[12] Brazma A., Schlitt T. Reverse engineering of gene regulatory networks: a finite state linear model. Genome Biol. 2003; 4:1–31.

[13] Mori T., Akutsu T. Attractor detection and enumeration algorithms for Boolean networks. Comput. Struct. Biotechnol. J. 2022; 20:2512–2520. 35685366 10.1016/j.csbj.2022.05.027 PMC9157468

[14] Hagber A., Schult D., Swart P. Exploring network structure, dynamics, and function using NetworkX. Proceedings of the 7th Python in Science Conference. 2008; 11–15.

[15] Mulyalin D. N2G Library, version 0.3.3. 2024; (28 March 2024, date last accessed) https://github.com/dmulyalin/N2G.

[16] yEd Works GmbH yEd Graph Editor, version 3.23.2. 2024; (28 March 2024, date last accessed) https://www.yworks.com/products/yed.

[17] Melkus G., Cerans K., Freivalds K., Zajakina D., Viksna J. Behavioral dynamics of bacteriophage gene regulatory networks. J. Bioinform. Comput. Biol. 2022; 20:225021. 10.1142/S0219720022500214 36102744

[18] McAdams H., Shapiro L. Circuit simulation of genetic networks. Science. 1995; 269:650–656. 7624793 10.1126/science.7624793

[19] Schlitt T., Brazma A. Current approaches to gene regulatory network modelling. BMC Bioinformatics. 2007; 8:S9. 10.1186/1471-2105-8-S6-S9 PMC1995542 17903290

[20] Oppenheim A., Kobiler O., Stavans J., Court D., Adhya S. Switches in bacteriophage lambda development. Annu. Rev. Genet. 2010; 39:409–429. 10.1146/annurev.genet.39.073003.113656 16285866

[21] Campbell A. Comparative molecular biology of lambdoid phages. Annu. Rev. Microbiol. 1994; 48:193–322. 7826005 10.1146/annurev.mi.48.100194.001205

[22] Hendrix R., Casjens S. Calendar R. Bacteriophage λ and its genetic neighborhood. The Bacteriophages. 2006; Oxford Oxford University Press 409–447.

[23] Paolozzi L., Ghelardini P. Calendar R. The bacteriophage Mu. The Bacteriophages. 2006; 2nd edn. Oxford Oxford University Press 469–496.

[24] Robb M., Shahrezei V. Stochastic cellular fate decision making by multiple infecting lambda phage. PLoS One. 2014; 9:e103636. 25105971 10.1371/journal.pone.0103636 PMC4126663

[25] Trinh J., Szekely T., Shao Q., Balaszi G., Zeng L. Cell fate decisions emerge as phages cooperate or compete inside their host. Nat. Commun. 2017; 8:14341. 28165024 10.1038/ncomms14341 PMC5303824

[26] Shao Q., Trinh J., Zeng L. High-resolution studies of lysis–lysogeny decision-making in bacteriophage lambda. J. Biol. Chem. 2019; 294:3343–3349. 30242122 10.1074/jbc.TM118.003209 PMC6416446

[27] Cheng H., Zheng Z., Cheng T. New paradigms on hematopoietic stem cell differentiation. Protein Cell. 2020; 11:33–44. 10.1007/s13238-019-0633-0 PMC6949320 31201709

